# Energy Absorption Characteristics of Composite Material with Fiber–Foam Metal Sandwich Structure Subjected to Gas Explosion

**DOI:** 10.3390/ma17071596

**Published:** 2024-03-31

**Authors:** Baoyong Zhang, Jin Tao, Jiarui Cui, Yiyu Zhang, Yajun Wang, Yingxin Zhang, Yonghui Han, Man Sun

**Affiliations:** Department of Safety Engineering, Heilongjiang University of Science and Technology, Harbin 150022, China; byzhang1982@163.com (B.Z.); 19835127449@163.com (J.C.); m18846190121@163.com (Y.Z.); 2006800307@usth.edu.cn (Y.W.); zhangyingxin01@163.com (Y.Z.); hanyonghui1@usth.edu.cn (Y.H.); 18745000523@163.com (M.S.)

**Keywords:** gas explosion, core layer, foamed metal, explosion-proof capability, composite

## Abstract

Based on the previous research on the energy absorption of foam metal materials with different structures, a composite blast-resistant energy-absorbing material with a flexible core layer was designed. The material is composed of three different fiber materials (carbon fiber, aramid fiber, and glass fiber) as the core layer and foamed iron–nickel metal as the front and rear panels. The energy absorption characteristics were tested using a self-built gas explosion tube network experimental platform, and the energy absorption effects of different combinations of blast-resistant materials were analyzed. The purpose of this paper is to evaluate the performance of blast-resistant materials designed with flexible fiber core layers. The experimental results show that the composite structure blast-resistant material with a flexible core layer has higher energy absorption performance. The work performed in this paper shows that the use of flexible core layer materials has great research potential and engineering research value for improving energy absorption performance, reducing the mass of blast-resistant materials, and reducing production costs. It also provides thoughts for the research of biomimetic energy-absorbing materials.

## 1. Introduction

Gas and other dangerous gas explosions are common hazards in industrial and mining enterprises. The key to rescue and relief is to reduce casualties after the explosion. Currently, explosion suppression methods can be broadly divided into gas explosion suppression, liquid explosion suppression, and solid explosion suppression [1].

Gas explosion suppression mainly employs experimental and numerical simulation methods from a macroscopic point of view to study the role of a single or complex gas explosion suppression system. The research objects are mainly inert gases, N_2_, CO_2_, and other gases. Liquid explosion suppression generally involves the addition of aqueous media by changing the spray molecular volume, additives, and charge of fine water mist on the gas explosion pressure and flame propagation velocity inhibition effect. In their research on solid explosion suppression, many scholars have found that porous materials have unique physical and mechanical properties and excellent energy absorption performance. They have carried out extensive research, and these materials have been widely used in the field of engineering protection.

Foam metal is one of the research hotspots of scholars due to its advantages of low density, large specific surface area, and high thermal conductivity. Zhuang et al. [2] conducted an experimental study on the suppression effect of different porous materials on the explosion of combustible gases. The results showed that the thickness and pore size changes in the composite porous material have a great influence on the explosion pressure and explosion intensity. Varun et al. [3] conducted a numerical simulation of three samples with different porosities (30%, 60%, and 80%) uniformly compressed in the uniaxial (Z-axis) direction to study the influence of porosity on the mechanical properties of open-pore Voronoi foam. Wang Yajun et al. [4] found through their personally designed experimental device that when the volume density of foam metal is higher, its explosion-proof performance is better, but adding a certain amount of coal dust to the explosion device will reduce the explosion-proof performance of foam metal. Wei Chunrong et al. [5] used a self-designed and processed square explosion experimental pipeline with a cross section of 30 cm × 30 cm to compare the explosion-proof effects of metal wire mesh, foam ceramics, and foam iron–nickel metal with different parameters. Yu Minggao et al. [6] studied the influence law of the synergistic effect of ultra-fine water mist and foam metal on explosion overpressure and found that changing the parameters, such as material porosity, can improve the explosion-proof effect of the experiment. Zhang Baoyong et al. [7] conducted an experimental study on the energy absorption performance of explosion-proof materials with a sawtooth structure and analyzed the influence of surface structure on energy absorption performance.

In addition to researching foam metal as a barrier material, scholars have also increasingly focused their attention on multilayer sandwich structures [8,9,10,11,12,13,14,15,16,17]. Tarlochan, F. [18] discussed the use of sandwich structures in energy absorption applications and found that sandwich structures are a good choice for energy absorbers. It is suggested that the way forward is to design sandwich structures by using a combination of “artificial intelligence/data mining and topology optimisation.” Many scholars have used sandwich structures designed with foamed metal in order to obtain higher energy absorption performance [3,19,20,21,22,23,24,25,26,27,28,29,30,31]. Zunjarrao, K. [32] reviewed current research on innovative sandwich structures, including integral woven corrugated cores, honeycomb cores, foam cores, and 3D printed core structures, and highlighted their versatility. Mao [33] used theoretical and numerical methods to study the attenuation of shock waves generated by gas explosions by an energy absorption device composed of aluminum foam and steel plates. The results showed that the multilayer composite structure has a good ability to reduce explosion load and attenuate air shock wave overpressure. The foam aluminum layer has the ability to attenuate explosion pressure before compression, and the attenuation ability decreases after the foam aluminum is completely compressed. Zhang et al. [34] studied and numerically analyzed the "effective" compressive strength and the dynamic response of corrugated sandwich panels with unfilled and foam-filled sinusoidal corrugated cores. The dynamic response of fully supported sandwich panels with unfilled and foam-filled sinusoidal corrugated cores under impact loading was analyzed using the finite element method. Chen et al. [35] carried out a study of the explosion protection properties of a composite structural barrier material with polymer interlayers using the LS-DYNA software and calibrated numerical models to simulate the explosion resistance and energy absorption capacity of the composite material under long-distance explosive loading conditions. Zhou et al. [36] predicted the compressive strength and dynamic response of a corrugated sandwich panel consisting of a panel and a metal foam core in close proximity to explosions. An energy-based method was proposed to predict the depth and scope of deformation in the outer panel, providing insights for the design and sizing of the core layer material. Langdon et al. [37] classified the energy absorption properties and applications of fiber-reinforced polymer composites and studied the multilayered (including the sandwich structure) and mixed composite metal structure materials. The numerical predictions were compared and analyzed with experimental data. In order to improve the protective performance of aluminum foam sandwich composites against blast shock wave and fragmentation penetration, Zhou et al. [38] studied the damage modes and mechanisms of the structure using the “blast + fragmentation” intrusion experiments. The effects of core material combination and blast distance on protective performance were also discussed and verified by the LS-DYNA numerical simulation. Santosa et al. [39] investigated the blast resistance and barrier performance of metal foam sandwich panels with different thicknesses, materials, and bulk densities by changing the impact distance between the blast load and the foam sandwich panels. Guo et al. [40] designed a metal foam-filled sandwich cylinder (MFSC) and found that adjusting the thickness of the foam based on the impact distance of the foam sandwich panels had no significant effect on the barrier performance by means of experiments and finite element calculations. In contrast, a finite element calculation found that adjusting the ratio of foam thickness to tube wall thickness can improve the load-carrying capacity and energy-absorption capacity of the energy-absorbing structure.

Inspired by the ancient Chinese philosophical idea of “combining hardness and softness” and the “sandwich” core structure, and based on the previous experimental study on the energy absorption characteristics of different explosion-proof surface materials, a composite explosion-proof and energy-absorbing material with foam metal as the upper and lower panels and fiber material as the core layer was designed. The explosion overpressure, flame propagation speed, flame temperature, and other characteristic parameters of the front and rear ends of the composite material after being subjected to a methane–air mixture gas explosion were collected and analyzed. The research results are expected to provide an experimental and analytical basis for the application of fiber–foam metal sandwich structure composite materials in the fields of gas explosion isolation and energy absorption.

## 2. Experimental

### 2.1. Materials

The experimental materials included foamed metal ferronickel, carbon fiber, aramid fiber, and glass fiber. The foam iron–nickel metal was produced by Jilin Zhuoer Metal Material Preparation Company (Jilin City, China). Pore density was 30, bulk density was 0.4–0.5 g/cm^3^, tensile strength was not less than 50 MPa, and compressive strength was not less than 250 kPa.

The fiber materials were all made of tough fiber fabrics from Zhongfu Fiber Textile Preparation Co., Ltd. (Suqian City, China) The fabric density was 1000 D, and the single-layer thickness was 1 mm. Five layers of each of the three materials, carbon fiber, aramid fiber, and glass fiber, were selected, and the core thickness was 5 mm.

### 2.2. Equipment

**Figure 1:**
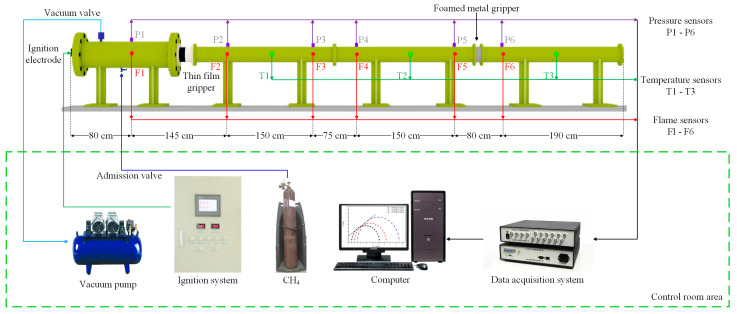
Experimental system.

As shown in Figure 1, the experimental system consists of six main parts in turn: the gas distribution system, ignition system, explosion chamber, explosion diffusion line, material gripper, and data acquisition system [41]. The end of the explosion chamber and the diffusion line are equipped with a PTFE film with a thickness of 0.3 mm and a breaking pressure of 90 kPa, and the edge of the gripper is sealed by a rubber ring, which is used to ensure the hermeticity inside the chamber. The main equipment of the gas distribution system is a vacuum pump. Under normal temperature and pressure conditions, the vacuum pump extracts part of the air from the explosion chamber to form a relative negative pressure state in the chamber. The required combustible gas is filled into the explosion chamber in this state. According to the experimental requirements, a certain volume of pure methane gas is charged to make it a mixed gas with an explosion concentration of 9.5%. An electric spark generator with an ignition voltage of 220 V and an ignition energy of 440 J is installed at the front end of the explosion chamber. The ignition electrode is responsible for remote charging and ignition by the terminal ignition system. The back end of the explosion chamber is sealed by a circular steel plate with a diameter of 300 mm and a thickness of 40 mm, and there is a hole of 118 mm in the center of the circular steel plate to connect with the diffusion line at the back end. The inner diameter of the diffusion pipe is 118 mm. It is spliced together from three hollow steel pipes with a single pipe length of 2200 mm. The total length of the explosion diffusion pipe is 6600 mm.

Flame sensors and pressure sensors, numbered 1–6, and temperature sensors, numbered 1–3, are installed at different locations in the experimental pipeline. The pressure sensor, flame sensor, and temperature sensor are all provided by Chengdu Tester Company (Chengdu, China), of which the pressure sensor model is CT100T, with a sensor range of 0~2 MPa; the flame sensor model is CKG100, with a response spectrum of 450 nm~980 nm; the temperature sensor model is C2 fast-response thermocouple, with a sensor range of 0~2500 °C; and the response time of all three sensors is less than 100 μs. Foam metal is installed in the material gripper, which is located between the No. 5 and No. 6 flame sensors and pressure sensors and is 0.15 m away from the No. 5 sensors. The data acquisition system used is the TST6300 data acquisition system from Chengdu Tester Company. The data acquisition objects are flame propagation speed, explosion overpressure, and flame temperature. The data sampling frequency is 100 kHz. The acquisition method is internal triggering. The recording time starts from the trigger ignition, and the entire recording process is less than 2 s.

### 2.3. Experimental Steps and Program

This experiment uses the explosion tube network device shown in Figure 1 to test the explosion-resistant performance of materials with different blast-facing structures by means of a gas explosion. The specific experimental process is as follows: First, clean the explosion chamber and explosion pipe network, seal the connection between the explosion chamber and the explosion-proof film holder with a 0.3 mm thick film, and seal the edge of the holder with a sealing ring around the explosion-proof film holder. Subsequently, the explosion chamber with a length of 1600 mm and a diameter of 300 mm is filled with a certain volume of methane gas, so that the interior of the chamber is filled with a methane–air gas mixture with a volume fraction of 9.50% for the explosion experiment. During the whole experiment, the data acquisition system collects data from different sensors at each measuring point for comparison, so as to observe the changing patterns of the impeded change in flame temperature propagation, the change in explosion overpressure before and after the material, and the flame propagation in the pipeline as an indicator to judge the explosion-resistant performance of the material.

The experimental conditions are shown in Table 1. The front and rear panels of the explosion-proof material are both foam iron–nickel metal. The front panel is surface-modified according to the experimental design requirements. Before the experiment, the wire cutting method is used to prepare the explosion-facing surface into a sawtooth wave with a thickness of 5 mm and an angle of 30°. The fiber core layer is divided into three working conditions: carbon fiber, aramid fiber, and glass fiber. In order to improve the data comparison effect of each working condition, experiment 1 is set as the control experiment. The thickness of the front panel is 15 mm, and no fiber material is added in the middle. The material parameters of other experiments are based on Table 1. In addition, in order to test the stability of the experimental platform, an empty pipe experiment without explosion-proof material is added.

The experiment is mainly conducted using the three aspects of the research data—the explosion overpressure, flame propagation speed, and flame temperature—as well as a comparative evaluation of different experimental materials on the explosion overpressure and other parameters of inhibition. The explosion overpressure, flame propagation, speed, and flame temperature-related formulas [41] are shown in (1)–(5):

(1) $$V=dp/dt=\left(p_{\max}-{\;p}_{i}\right)/\Delta t=\Delta p/\Delta t$$

where *V* is the rate of decline in overpressure, in MPa/s; *P*_max_ is the front end of the test material maximum explosion overpressure, in MPa; *P*_i_ is the back end of the test material maximum explosion overpressure, in MPa; Δ*p* is the test material before and after the two ends of the explosion pressure difference, in MPa; and Δ*t* is the sensor detection signal time difference, in seconds.

(2) $$\zeta =\left(p_{\max}-p_{i}\right)/p_{\max}$$

where *P*_max_ is the experimental material in the front channel of the maximum overpressure, in MPa; *P*_i_ is the explosion conditions of the experimental material at the back end of the maximum explosion overpressure, in MPa; and *ζ* is the overpressure attenuation rate, that is, the material of the maximum overpressure reduction control ability.

The blocking effect of the experimental material on the flame propagation velocity can be compared with the flame propagation velocity decay rate as follows:

(3) $$\mu =\Delta {\nu /\nu }_{\max}$$

where *μ* is the foam metal before and after the flame propagation velocity attenuation rate, in m/s; Δ*ν* is the foam metal before and after the difference in velocity propagation, in m/s; and *ν*_max_ is the foam metal during the explosion before the end of the maximum value of velocity, in m/s.

The damping effect of the experimental materials on the flame temperature can be compared with the flame temperature decay rate as follows:

(4) $$\eta =\left(T_{f,\max}-T_{i,\max}\right)∕T_{f,\max}$$

where *T*_max_ is the maximum temperature at the front end of the experimental material, in °C; *T*_i_ is the maximum temperature at the back end of the experimental material, in °C; and *η* is the flame temperature attenuation rate, i.e., the material’s abatement control ability for the maximum temperature.

The overall protection effect of the experimental materials can be compared with the explosion quenching parameters as follows:

(5) θ=TP

where θ is the quenching parameter, in MPa·°C; *T* is the flame temperature, in °C; and *P* is the explosion overpressure, in MPa.

## 3. Results and Discussion

### 3.1. Comparative Study of the Blast Overpressure Barrier Effect

**Figure 2:**
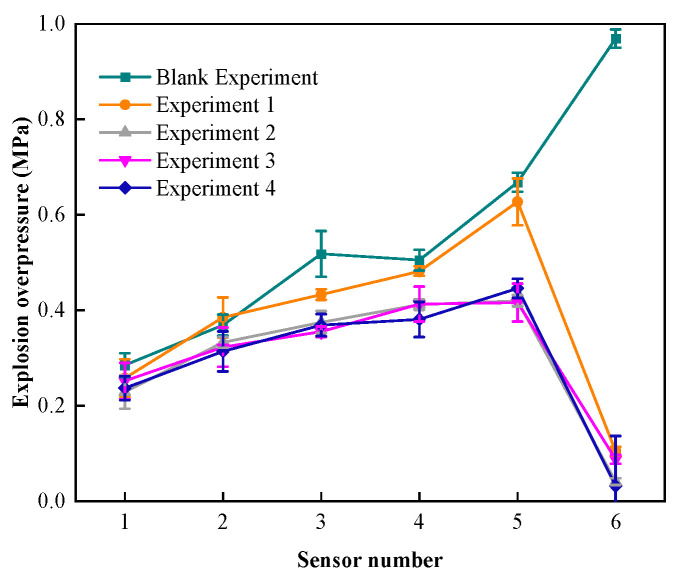
Explosive overpressure–distance data.

Figure 2 shows the explosion overpressure–distance data before and after different experiments. The maximum explosion overpressure of experiments 1 to 4 is reduced to 0.105 MPa, 0.067 MPa, 0.069 MPa, and 0.034 MPa, respectively, after passing through the explosion-proof material. The sensor value is significantly reduced after the explosion-proof material, and it can be clearly seen from the experimental data that experiments 2 to 4 have a better pressure drop effect under the same gas explosion overpressure propagation conditions [41] and are better than the single foam metal structure explosion-proof material.

At the same time, the overpressure attenuation results of the three fiber material core explosion-proof materials have large differences: the overpressure attenuation rate of the carbon fiber–foam metal explosion-proof material to gas explosion overpressure is 85.27%, and the explosion overpressure before and after the material is reduced by 0.388 MPa. The overpressure attenuation rate of aramid fiber–foam metal explosion-proof material to gas explosion overpressure is 84.24%, and the explosion overpressure before and after the material is reduced by 0.369 MPa. The overpressure attenuation rate of glass fiber–foam metal explosion-proof material is 92.01%, and the explosion overpressure before and after the material is reduced by 0.392 MPa. From the perspective of the overpressure attenuation rate of different core layer explosion-proof materials, glass fiber has the best effect, followed by aramid fiber, and carbon fiber has the lowest effect.

**Figure 3:**
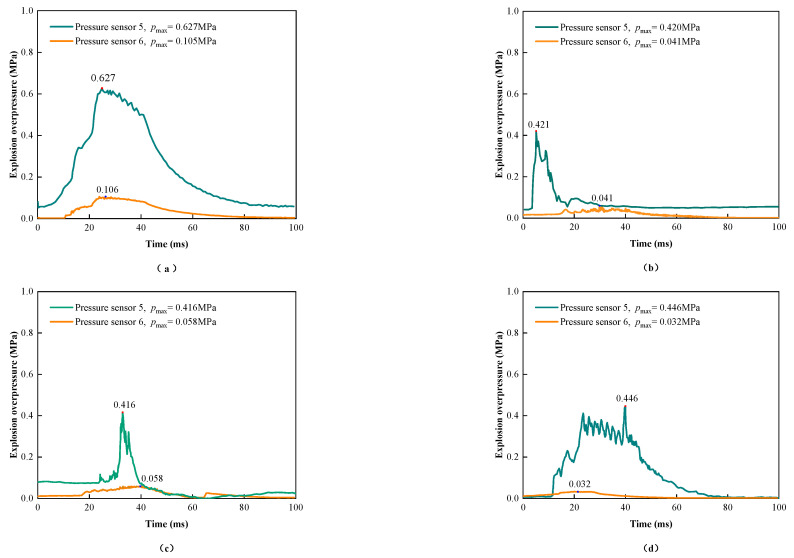
Different composite material overpressure change rule with time. (**a**) Without a core layer. (**b**) Carbon fiber core layer. (**c**) Aramid fiber core layer. (**d**) Glass fiber core layer.

The evolution of explosion overpressure before and after different composite materials is shown in Figure 3. According to the data analysis in the figure, the explosion overpressure drop rates of experiments 1 to 4 are 11.82 MPa/s, 3.62 MPa/s, 4.11 MPa/s, and 2.78 MPa/s, respectively. There are two reasons why the explosion overpressure drop rates of experiments 2 to 4 are smaller than for experiment 1: (1) the maximum explosion overpressure detected by sensor P5 in experiments 2 to 4 is smaller than that of experiment 1; (2) due to the addition of tough fibers, the material hinders the propagation of the explosion shock wave, prolongs the time for the explosion shock wave to pass through the entire material, and thus plays a role in weakening the penetration ability of the explosion shock wave [41].

Through analysis, it was concluded that when the explosion shock wave propagates inside the foam metal, due to the porous structure characteristics of the foam metal itself, the shock wave overpressure can be divided into several small parts when it passes through the foam metal material, and the pressure can be quickly transmitted and absorbed, reducing the propagation speed of the shock wave and achieving the effect of blocking the propagation of the explosion overpressure [41]. This experiment increases the explosion-proof performance of foam metal under explosion overpressure by adding fiber materials inside the foam metal so as to further weaken the explosion shock. When the explosion overpressure hits the fiber material, due to the fact that the fiber material is a flexible material with better deformation ability, the fiber material will deform and play a buffering role in the process of being subjected to the explosion shock, further protecting the rear material from the stress it bears, improving the overall energy absorption characteristics of the material, and further reducing the explosion shock at the rear of the material. Under the premise of the same foam metal as rigid support and the same surface porosity, glass fiber has higher fiber density, better anti-deformation ability, and absorbs more shock wave overpressure released by gas explosions.

### 3.2. Comparative Study of the Flame Propagation Blocking Effect

**Figure 4:**
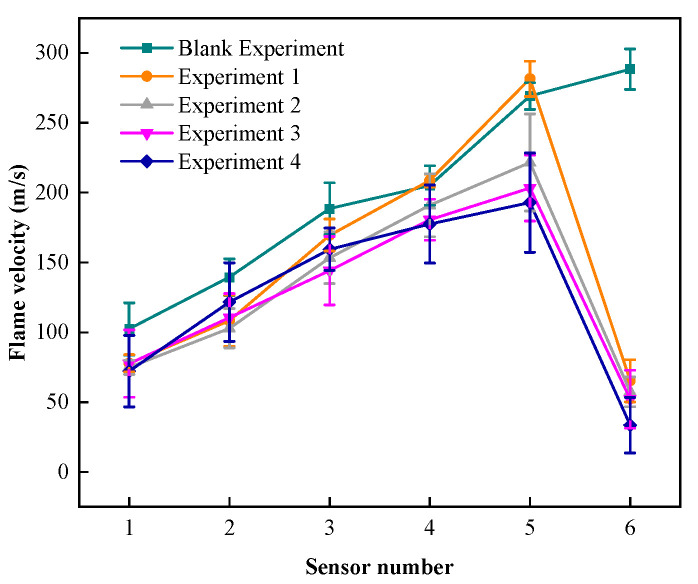
Flame speed–distance data.

The relationship between flame propagation speed and propagation distance is shown in Figure 4. In experiments 1 to 4, the speeds detected by the sensors at the rear of the explosion-proof material are 65.28 m/s, 57.32 m/s, 52.18 m/s, and 33.48 m/s, respectively. After calculation, the flame propagation speed decreases by 216.27 m/s, 164.17 m/s, 151.09 m/s, and 160.30 m/s, respectively. The results show that adding a fiber material core layer to the foam iron–nickel panel can absorb and suppress the further propagation of the flame, thereby achieving the purpose of improving the explosion-proof effect. The reason for this is that when the flame passes through the fibers inside the foam metal, a “channel” that is smaller and denser than the original foam metal is formed. When the flame flows in the denser “channel,” the oxygen supply of the flame is further blocked, achieving the purpose of more easily isolating the flame propagation [41,42]. Similar to the experimental results of explosion overpressure suppression, glass fiber core layer material has the best flame propagation suppression effect.

**Figure 5:**
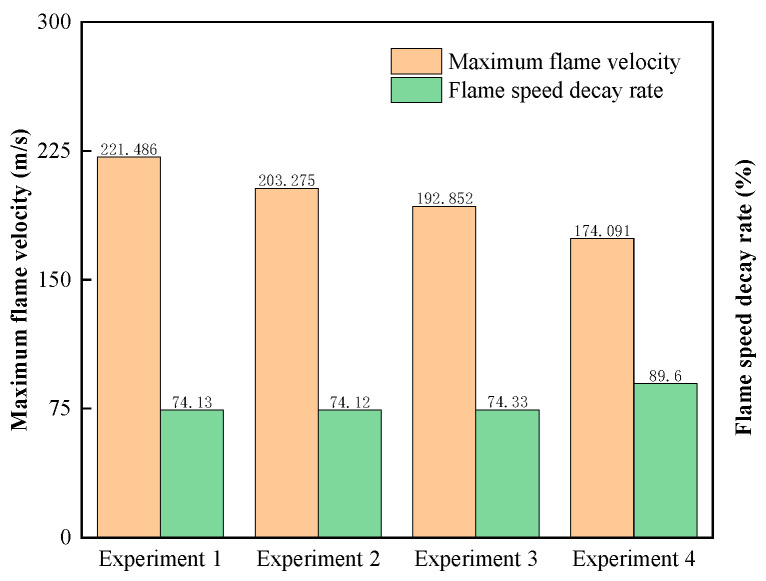
Flame velocity suppression effect data.

Figure 5 shows a graph of the flame propagation velocity suppression effect data. The flame propagation velocity detected at the rear end of the flame retardant material was reduced for all conditions, with the attenuation rate ranging from 74.12% to 89.60%.

The flame propagation speed attenuation rate of the explosion-proof material before and after experiment 4 is 89.60%, the maximum flame propagation speed is 174.091 m/s, and it has the best blocking effect on the explosion flame propagation. The flame propagation speed attenuation rates of experiments 1 to 3 are similar, at 74.13%, 74.12%, and 74.33%, respectively. The maximum flame propagation speeds are 221.486 m/s, 203.275 m/s, and 192.852 m/s, respectively. The maximum flame propagation speeds of experiments 2 to 4 are all lower than for experiment 1 to different degrees, which indicates that the protection effects of experiments 2 to 4 are all better than experiment 1 under the impact of flame.

**Figure 6:**
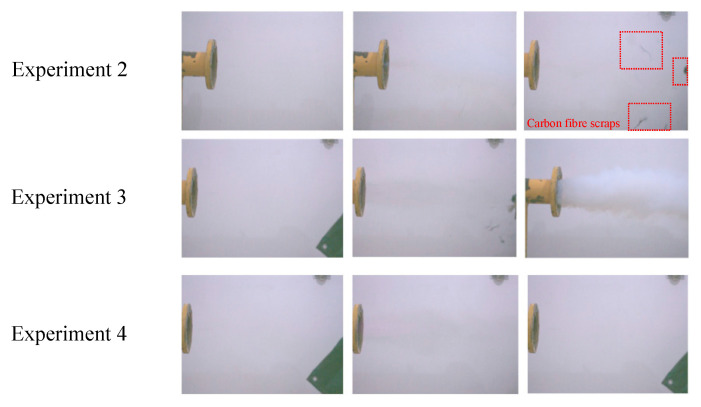
Fire propagation effect diagram at the end of the explosion pipe network.

Figure 6 shows the flame propagation effect diagram at the end of the explosion pipeline. It can be seen from the pictures of experiment 4 that only part of the smoke and gas are diffused from the end of the pipeline, and there is no situation where the material or flame rushes out of the end of the explosion pipeline. The experimental effect is the best. In experiment 3, only a large amount of smoke was sprayed out under the premise of ensuring the integrity of the experimental material. The experimental effect is better. In experiment 2, a small amount of carbon fiber material was sprayed out of the pipeline along with smoke, and the integrity of the core material was not guaranteed. The experimental effect is the worst. By comparing the explosion overpressure and flame propagation results of each working condition and combining the analysis of the actual effect at the end of the experimental pipeline, it is once again demonstrated that adding tough fiber materials to foam metal can effectively improve the overall explosion-proof performance of the material compared to single foam metal experiments.

### 3.3. Comparative Study of the Flame Temperature Barrier Effect

**Figure 7:**
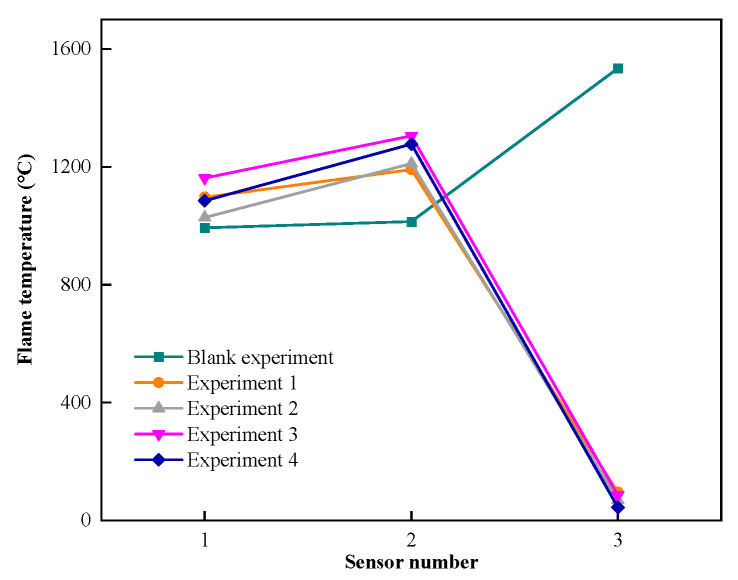
Flame temperature–distance data.

Figure 7 shows the flame temperature–distance data before and after different explosion-proof materials. As can be seen from the figure, the overall trend of flame temperature propagation is similar to that of explosion overpressure and flame propagation speed. After adding flexible fiber materials to the foam metal material, the explosion-proof effect is better than that of a single foam metal material. Glass fiber has the best effect on the explosion-proof material with a fiber core layer, followed by aramid fiber, and carbon fiber has the worst effect on the explosion-proof material.

The temperature values detected at the rear of the explosion-proof materials in experiments 1 to 4 are 96.112 °C, 69.578 °C, 85.770 °C, and 44.875 °C, respectively. The flame temperature drop amplitudes are 1094.928 °C, 1141.572 °C, 1219.490 °C, and 1232.695 °C, respectively. The temperature attenuation rates are 91.93%, 94.26%, 93.43%, and 96.49%, respectively. It can be seen from the experimental results of flame temperature attenuation and temperature change that the explosion-proof materials composed of three different fiber core layer materials are still slightly better than the single foam metal material in terms of flame temperature suppression, and the experimental results of the glass fiber core layer are still the best.

**Figure 8:**
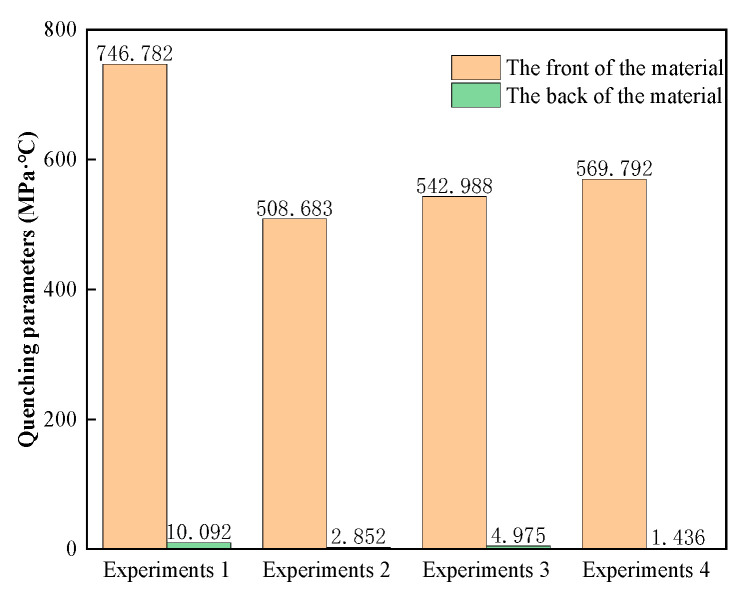
Extinction parameter data at the front and rear of the material.

Figure 8 shows the comparison of the extinction parameters of different explosion-proof surfaces. The extinction parameter at the rear of the explosion-proof material in experiment 4 is 1.53 MPa·°C, and the explosion-proof effect is the best. The extinction parameters at the rear of the explosion-proof materials in experiments 1 to 3 are 10.09 MPa·°C, 4.66 MPa·°C, and 7.63 MPa·°C, respectively. The values of the extinction parameters before and after the materials are 755.75 MPa·°C, 546.41 MPa·°C, 564.07 MPa·°C, and 542.72 MPa·°C, respectively. When the extinction parameter is lower than 390 MPa·°C, people and equipment are within the relative safety limit, and the lower the extinction parameter, the better the protection effect. Data analysis shows that experiment 1 has a larger extinction parameter drop because the explosion-proof material has a larger initial explosion overpressure value at the front end, which leads to an increase in the extinction parameter. The extinction parameter values at the rear of the materials in experiments 2 to 4 are lower than in experiment 1, indicating that this core layer design can more effectively reduce the damage to the protected target caused by explosion shock, flame, and temperature.

### 3.4. Analysis of the Effect of Different Composite Materials to Prevent the Explosion

**Figure 9:**
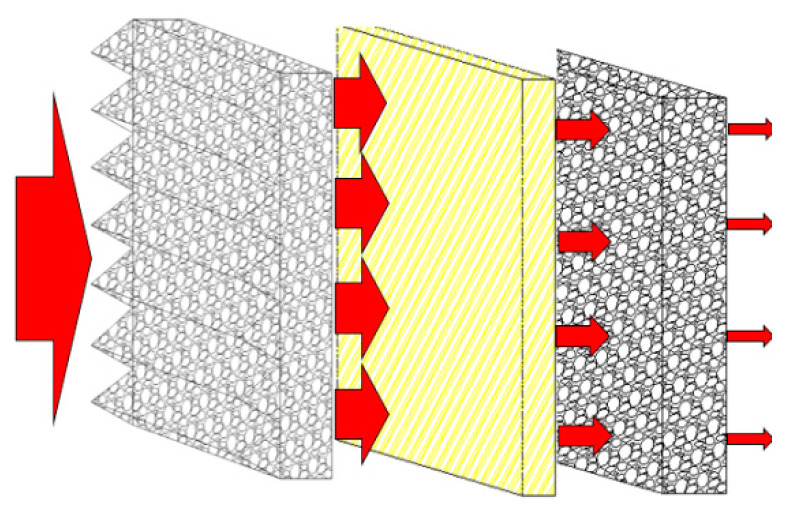
Schematic diagram of the fiber–foam metal core structure composites.

Experimental studies have found that adding different types of fiber materials to foam metals can effectively improve the overall explosion-proof performance of the experimental materials. Figure 9 shows a schematic diagram of a fiber–foam metal core structure composite material. When the explosion shock wave and flame impact the explosion-proof surface of the foam metal, the explosion and flame impact will penetrate the interior along the sawtooth of the explosion-proof surface. The explosion shock wave and flame are highly concentrated in this area and cause deformation, energy reflection, and scattering at the wall surface when entering the foam metal, thereby achieving the effect of making the material explosion-proof. Adding different types of fiber materials as the core layer in the material can form a “channel” that is smaller and denser than the original foam metal fine pores, which increases the propagation path, further inhibits the occurrence of chemical reactions, and buffers the foam metal while the deformation makes the experimental material absorb more energy holistically, thereby achieving the purpose of improving the explosion-proof effect.

In the experiment, the overall explosion-proof effect of adding glass fiber to foam metal is better than that of other fiber material experiments. The preliminary analysis shows that the fiber density of glass fiber material is higher, so the overall explosion-proof effect of glass fiber is better.

**Figure 10:**
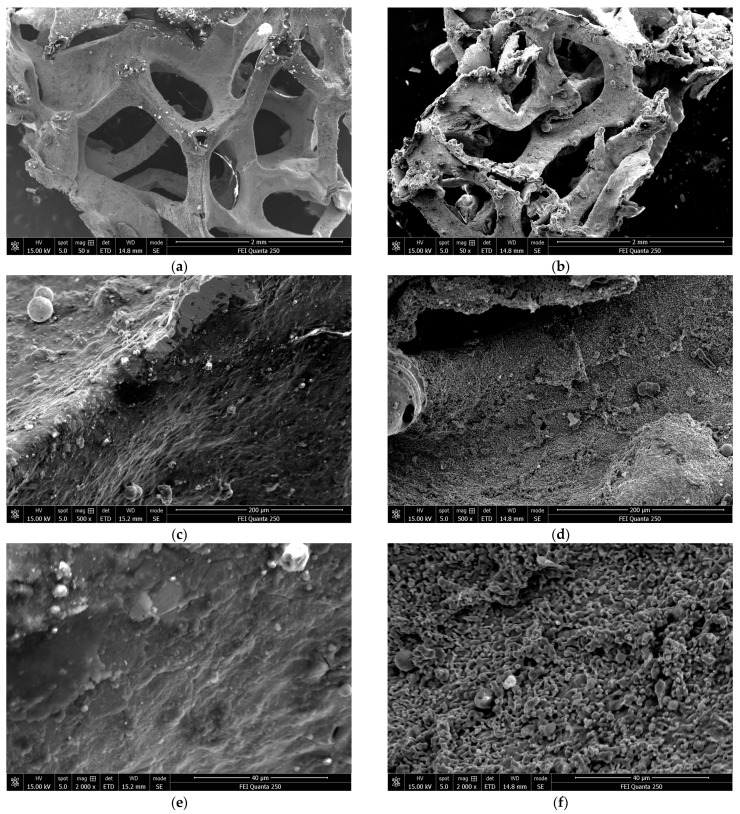
Scanning electron microscope images of foamed metal materials at different magnifications. (**a**) Pre-experiment (50×). (**b**) Post-experiment (50×). (**c**) Pre-experiment (500×). (**d**) Post-experiment (500×). (**e**) Pre-experiment (2000×). (**f**) Post-experiment (2000×).

In order to observe more intuitively the state of the foamed FeNi metal before and after the passage of the overpressure shockwave and flame, a scanning electron microscope (SEM) was used to take pictures of the foamed metal before and after the experiments (Figure 10). The changes in the foamed metal material in the microscopic view can clearly be distinguished, which will help with future research on energy absorption in foamed metal. A comparison of the images shows that the surface structure of the foamed metal received damage and became rougher after the shockwave overpressure and the passage of the flame, along with traces of cracking, melting, ablation, and material detachment, which changed the pore structure of the foamed metal. When the surface of the foamed metal was observed at magnification, the surface changes were evident after the passage of the flame, presumably as a result of oxidation or other chemical reactions.

## 4. Conclusions

This experiment used a self-designed explosion tube network experimental platform to study the energy absorption performance of three different fiber core layer blast-resistant composite materials on methane–air mixture gas explosions. The energy absorption effect of blast-resistant composite materials with flexible fiber core layers is more significant than that of single foam metal structures. At the same time, the composite material using glass fiber as the core layer has stronger energy absorption performance than the composite materials using carbon fiber and aramid fiber and can provide better protection for the target. In short, by selecting different flexible core layer materials for research, it is helpful to reduce the production cost and reduce the overall weight of the material while ensuring that the overall energy absorption performance of the blast-resistant material is further improved. We believe that our research can not only provide experimental verification for the improvement of the energy absorption performance of foam metal structure blast-resistant materials, but also provide experimental solutions and theoretical analysis for the measurement of energy absorption performance. This study provides enlightenment for further optimizing the energy absorption performance and application of foam metal structures.

## 5. Patents

Patent application for research-related content: a kind of high-efficiency explosion-proof wall composite energy-absorbing and diffusion-resistant material, No. 202310992747.9.

## Figures and Tables

**Table 1:** Design parameters of external explosive surface tests.

Number of Test	Fiber Type	Foamed Metal	Body Density (g/cm^3^)	Serrated Thickness (mm)	Material Thickness (mm)
1	—	foamed ferronickel	0.5	5	15 + 0 + 10
2	carbon	foamed ferronickel	0.5	5	10 + 5 + 10
3	aramid	foamed ferronickel	0.5	5	10 + 5 + 10
4	glass	foamed ferronickel	0.5	5	10 + 5 + 10

## Data Availability

Data are contained within the article.
